# Interventions to increase circularity and reduce environmental impacts in food systems

**DOI:** 10.1007/s13280-023-01953-x

**Published:** 2023-11-16

**Authors:** Benjamin van Selm, Hannah H. E. van Zanten, Renske Hijbeek, Corina E. van Middelaar, Marijke Schop, Martin K. van Ittersum, Imke J. M. de Boer

**Affiliations:** 1 Animal Production Systems Group, Wageningen University & Research, P.O. Box 338, 6700 AH Wageningen, The Netherlands; 2 Plant Production Systems Group, Wageningen University & Research, P.O. Box 430, 6700 AK Wageningen, The Netherlands; 3 Farming Systems Ecology Group, Wageningen University & Research, P.O. Box 430, 6700 AK Wageningen, The Netherlands; 4 R&D monogastrics, Agrifirm, Landgoedlaan 20, 7325 AW Apeldoorn, The Netherlands; 5 Department of Crop Production Ecology, Swedish University of Agricultural Sciences, 75007 Uppsala, Sweden

**Keywords:** Circular food systems, Dietary change, FOODSOM, GHG emissions, Land use

## Abstract

Applying specific circularity interventions to the food system may have environmental benefits. Using an iterative linear food system optimisation model (FOODSOM), we assess how changes in human diets, imports and exports, and the utilisation of waste streams impact land use and greenhouse gas emissions (GHG). After including these circularity principles, land use and GHG emissions were on average 40% and 68% lower than in the current food system, primarily driven by a reduction in production volumes and a shift towards feeding the domestic population. Shifting from the current diet to a circular diet decreased land use with 43% and GHG emissions with 52%. Allowing up to half of each nutrient in the human diet to be imported, while balancing imports with equal exports in terms of nitrogen, phosphorus and potassium, also decreased land use (up to 34%) and GHG emissions (up to 26%) compared to no imported food. Our findings show that circularity interventions should not be implemented mutually exclusively; by combining a circular diet with imported food and fully utilising waste streams, the lowest land use and GHG emissions can be realised.

**Supplementary Information:**

The online version contains supplementary material available at 10.1007/s13280-023-01953-x.

## Introduction

Transitioning the current food system to a more circular one has been proposed as a promising pathway to reduce the environmental impacts of the food system (Jurgilevich et al. 2016; De Boer and Van Ittersum 2018; Billen et al. 2021). The current food system contributes to around one third of global greenhouse gas (GHG) emissions and occupies about 40% of the earth’s terrestrial surface (Foley et al. 2011; Crippa et al. 2021). Furthermore, food is currently inequitably distributed, the food system fails to ensure that all people receive access to affordable, healthy and nutritious food (Godfray et al. 2010). Circular food systems aim to safeguard natural resources by prioritising the use of biomass for basic human needs (e.g. food production) while avoiding the non-essential use of biomass and unnecessary losses (Muscat et al. 2021). Unavoidable losses, including food waste, are recycled in a circular food system as animal feed or fertiliser to better close nutrient cycles. By reducing crop production for animal feed or the use of artificial fertilisers, the environmental impacts of the food system can potentially be reduced (Van Zanten et al. 2018, 2019; van Selm et al. 2022).

Despite interest in the concept of circular food systems, little research has been done to quantitatively assess the environmental consequences of various circular food system principles (e.g. safeguarding natural resources, avoiding the non-essential use of biomass and unnecessary losses, prioritising biomass for basic human needs, and utilising and recycling by-products and waste streams, Muscat et al. (2021)). The quantification of such circular food system principles and concepts has mainly focused on the role of livestock in circular food systems and their ability to upcycle low opportunity cost biomass (i.e. by-products, food waste and grassland resources) into animal-sourced food (Van Hal et al. 2019; Frehner et al. 2022; van Selm et al. 2022).

Uncertainties in the design of more circular food systems include the role of imports and exports, changes in human diets and the utilisation of waste streams. Firstly, the import and export of food and raw materials (e.g. soybeans, wheat, milk powder) makes the closing of nutrient cycles in a circular food system challenging (Billen et al. 2014; Lassaletta et al. 2014; Smit et al. 2015; Koppelmäki et al. 2021), and the optimal geographical scale at which nutrient cycles should be closed in a food system is largely unknown (Koppelmäki et al. 2021), if existing. Secondly, transitioning from the current diet to a more circular diet is deemed necessary to safeguard natural resources and mitigate the environmental impact of the food system (Herrero et al. 2020; van Selm et al. 2022). More specifically, the environmental impact of the food system can be reduced by limiting animal-sourced food consumption (Poore and Nemecek 2018; Springmann et al. 2018; Willett et al. 2019). However, reducing animal-sourced food consumption relies on peoples' willingness to change behaviour and political will to steer changes in consumption patterns (Godfray et al. 2018), yet specific dietary compositions are debated. Thirdly, feeding some waste streams (e.g. food waste) to animals is currently prohibited in some countries due to food safety risks (zu Ermgassen et al. 2016; Salemdeeb et al. 2017). Prohibiting the feeding of waste streams to animals makes the recycling of unavoidable losses challenging (zu Ermgassen et al. 2016). Overcoming these challenges is seen as a promising strategy to reduce environmental impacts of animal production (Van Hal et al. 2019; Van Zanten et al. 2019; van Selm et al. 2022), but the size of the comparative environmental benefit is unknown. Our aim is to explore how future circular food systems could be designed to achieve minimal agricultural GHG emissions and land use using the Netherlands as a case study. We expect trends to be similar for other countries, especially affluent countries with a relatively high share of animal-sourced food consumption.

Transitioning from the current food system to a circular food system has been identified as a political priority by the Dutch government to reduce environmental impacts of agriculture (Ministry of Agriculture Nature and Food Quality 2018). Currently, the Netherlands is a highly productive agroecological area with high animal numbers sustained by imports of animal feed and exports of animal-sourced food (Smit et al. 2015; De Boer and Van Ittersum 2018; van Grinsven et al. 2019; Post et al. 2020). Consequently, manure and nutrient surpluses have contributed to biodiversity loss and GHG emissions. Taking the Netherlands as a case study, we explore the importance of three food system interventions leading to increased circularity: (1) reducing import and export of food, (2) dietary change, and (3) the use of waste streams in the food system.

For this analysis, we have developed an iterative linear optimisation model called FOODSOM (Food System Optimisation Model). FOODSOM minimises domestic land use and GHG emissions while meeting the dietary requirements of the Dutch population. Reducing agricultural land use can lead to more land being available for natural areas and biodiversity, while reducing GHG emissions can contribute to minimising the effects of climate change. Feeding the domestic population has a narrower focus than the current Dutch agricultural production, which is largely export-driven. Nonetheless, focusing on feeding the domestic population allows to, (1) compare current land use and GHG emissions from agricultural production with those associated with domestic consumption, (2) assess the comparative effectiveness of different circularity interventions, and (3) assess how the footprint of Dutch consumption patterns changes in a circular food system. To this end, twenty-four circular food systems designs and a reference scenario were compared with the aim to understand how the inclusion of different food system interventions can influence land use and GHG emissions.

## Materials and Methods

**Fig. 1:**
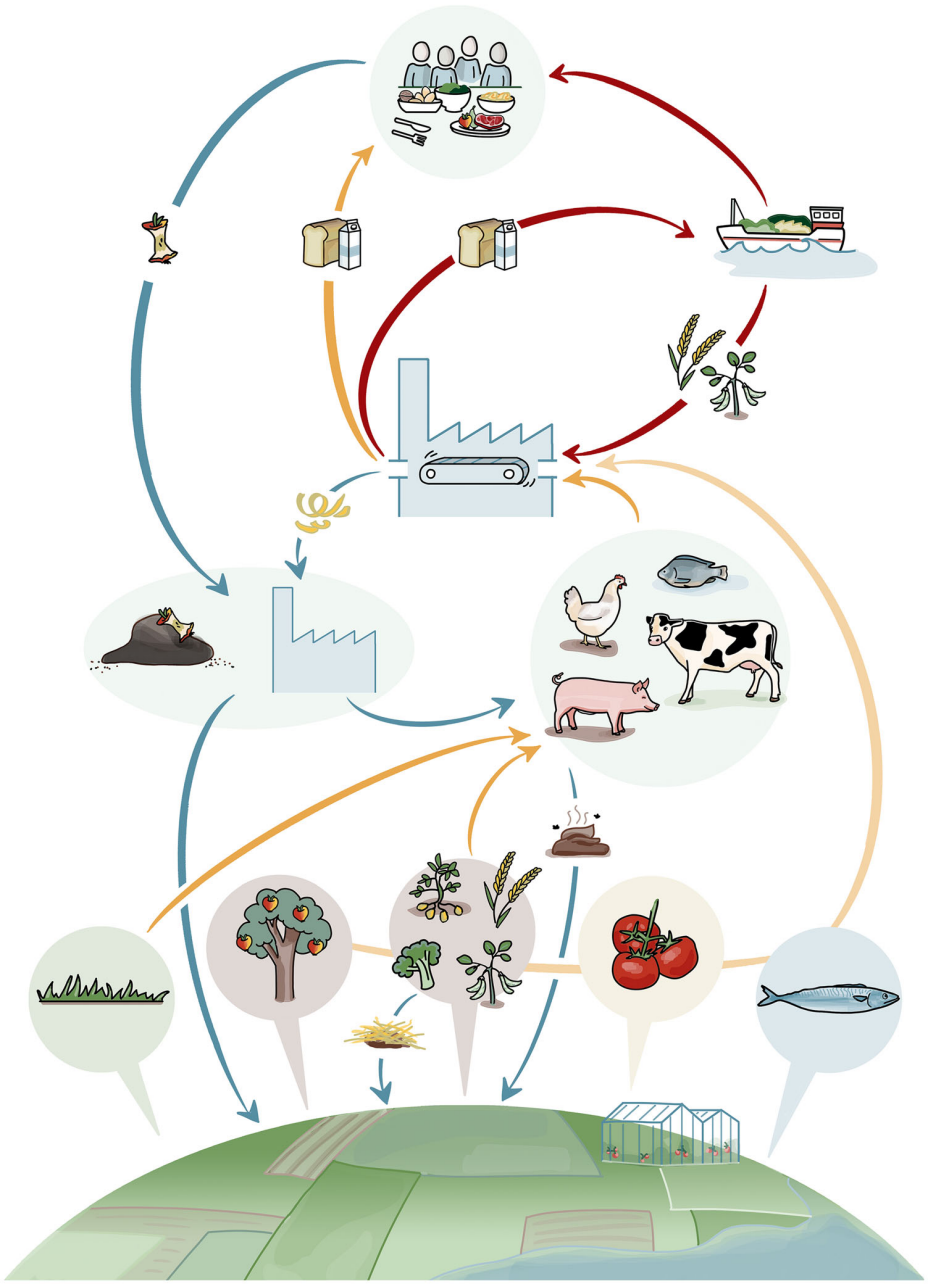
Framework of the FOODSOM model

In this study, FOODSOM was developed and employed to assess how circular food system interventions can achieve minimum agricultural land use and GHG emissions from food production and consumption in the Netherlands (Fig. 1). FOODSOM is an iterative linear optimisation model of a circular food system in the Netherlands created in GAMS 42.

### Land, crops and fertilisation

In the FOODSOM, agricultural land is split into three land use classes: annual cropland, grassland and permanent cropland. Annual cropland and grassland contain four soil texture classes: sand, clay, loam and peat. Peat land is assumed to be only suitable for growing grass and unsuitable for cultivating annual crops due to high groundwater levels in the Netherlands. The permanent cropland use category contains three infrastructure classes: greenhouses, orchards (e.g. apples, cherries, etc.) and mushroom sheds. The area of annual cropland, grassland and permanent cropland was fixed to current levels.

For the cultivation of arable land, FOODSOM can select from 49 crops. For the cultivation of permanent cropland, the options are limited. The model can select from four crops in greenhouses, six crops in orchards and one crop in mushroom sheds. In our study, crop yields are based on national statistics or survey data (CBS 2019; De Ruijter et al. 2020). Only one crop productivity level is included. In addition to the main crop yield, cereal and oil seed crops also produce a residue crop yield; residue yields are based on default data (PPO 2018).

Restrictions are placed on which crops can be grown on each land/soil or land/infrastructure combination based on current practice and expert knowledge (e.g. red cabbage can only be produced on arable clay or loam soils). Land/soil and land/infrastructure suitability applies to the entire country due to similar climatic conditions. Crop rotations in the FOODSOM are simulated using a maximum crop share, for example, if the minimum frequency a crop can be grown is every second year, the maximum area of the crop is 50% of the province, arable land and soil type combination area.

Crop fertilisation includes nitrogen (N), phosphorus (P), and potassium (K) fertilisation. A range of fertilisers are available in the model: artificial fertiliser, animal manure, compost and crop residues. In the food system designs, N fertilisation is calculated by accounting for harvested N, N losses from volatilisation (N_2_O, NH_3_, NO_x_, N_2_), N losses from leaching and N inputs including deposition, mineralisation in peat soils and biological N fixation from legume crops. In the reference scenario, N fertilisation is based on current legislation and accounts of the fertiliser replacement values (NFRVs) of organic amendments. The NFRV accounts for the availability of N in the organic amendments, relative to mineral fertiliser. Additional restrictions were placed on the application of animal manure to comply with N fertilisation legislation (RVO 2021). Phosphorus and K fertilisation are calculated using a balanced approach, and harvested P and K values are multiplied by an unavoidable loss fraction (12.5%) (Lun et al. 2018).

### Marine fisheries

Marine fisheries are a source of fish for human consumption, high-quality by-products for animal consumption or a source of nutrients for the soil (through composting). Current fisheries landing are assumed to be maintained using a five-year average (2014–2018) (CBS 2019).

### Livestock

In FOODSOM, livestock includes dairy cattle, beef cattle, pigs, broiler chickens and laying hens with three productivity levels (high, medium and low). Current livestock systems in the Netherlands are represented by the high productivity level. Parent stocks (e.g. sow in pig system) and reproduction stocks (e.g. heifer in a dairy system) of producing classes of livestock are also included FOODSOM.

For our study, livestock nutrient requirements are based on Van Hal et al. (2019). The nutrient requirements of livestock are fulfilled, depending on the circularity intervention, by feed crops (e.g. maize silage, wheat, etc.), grassland, by-products from food processing (e.g. wheat bran, sugar beet pulp, etc.) and food waste. Food waste cannot be consumed by ruminants due to specific food safety risks (zu Ermgassen et al. 2016). Livestock also produce slaughter waste as a by-product. The nutritional value of feed crops, grassland, by-products and food waste are obtained from the CVB (Spek and Van Wesemael 2021).

Aside from producing food for meat, milk and eggs, livestock also produce manure which can be used as a fertiliser for crops and grassland. In the model, all manure is captured in a manure management system except for that of grazing ruminants. Manure captured in manure management systems can be exported to other provinces to be applied on, for example, arable land. Grazing ruminants excrete manure directly onto grassland, the proportion of manure excretion onto grassland is a function of the model using the proportion of grazed grassland dry matter intake (i.e. if 50% of dry matter came from grazed grass then 50% of manure excretion is in grassland).

### Import & export

Food can be imported into the Netherlands and exported from the Netherlands. Total imports are restricted by the amount of each nutrient (e.g. calories, protein, vitamin A) in imported foods relative to the total consumption of each nutrient in the human diet. For example, in the 25% import intervention, up to 25% of each nutrient consumed could come from imported foods. The remaining 75% of each nutrient must come from domestically produced food. Each food item was assumed available (for import) in unlimited quantities.

Across the different scenarios, when food is imported, food is also exported. For all interventions, N, P, and K in imports must equal N, P, and K in exports. This nutrient balance is applied to plant-sourced food and animal-sourced food to ensure fair exchange of food items. However, the environmental impact of the imported food is unknown; neither land use nor GHG emission of imported food is considered. Instead, land use and GHG emissions from exported products remained allocated to the Netherlands. In the reference scenario imports and exports were based on current imports derived from the FAO food balance sheets (FAO 2019).

### Processing and transportation

Crop, fish and animal products require processing into human food, which results in by-products suitable as animal feed or soil amendments. In this study, fractions of human food and by-products are based on FAO technical conversion factors (FAO 1996).

It is assumed that crop and livestock products are processed into food, feed and by-products in the province of production and transported to the province of consumption after processing. Food, feed, by-products, food loss and waste, and manure can be transported between provinces in the Netherlands. FOODSOM includes the temporal aspect of food demand and availability on a monthly basis. Not all human food products are available all months of the year, and the availability of perishable food items for a given month is based on harvest dates and shelf life.

### Food waste

Food waste occurs at all stages along the supply chain including post-harvest, processing and packaging, distribution and retail, and consumption. Post-harvest and processing and packaging losses occur in the province of production, while distribution and consumption losses occur in the province of consumption. The percentage of food lost or wasted varies depending on the stage of the supply chain and type of product (Caldeira et al. 2019).

Due to the nature of the supply chain, different food waste stages are handled differently in the model. Post-harvest and processing and packaging losses are separated for animal feed or pooled into a single product for composting. Distribution and retail losses are pooled together into 11 products based on their food family (e.g. all grain products are pooled into a single grain waste product) -with differing nutrient compositions- for animal feed or pooled into a single product for composting. The nutrient composition is calculated using a weighted average of food items wasted. Consumption losses are pooled into a single product using a weighted average of food items wasted for animal feed or for composting.

### Human population and diet

National statistics are used to determine the size of the population per province in the Netherlands (CBS 2019). The population is split into eight age classes and two gender classes. Minimum and maximum nutrient requirements were included per age and gender class (Brink et al. 2019). In total the requirements of 27 nutrients are included in the model including macro- and micro-nutrients, and, vitamins (Table 1). Two diets are included, the current diet and a circular diet. Firstly, the current diet is based on a national consumption survey and included a fixed consumption per food group (van Rossum et al. 2020). In addition, nutrient contents of food items are based on the Dutch Food Composition Table (RIVM 2019). Secondly, the circular diet allows for variable consumption per food group. An upper limit for consumption is placed on each food group to ensure the circular diet remains feasible (Table 2). The upper limit of food groups that did not increase the risk of non-communicable diseases was based on the 95th percentile of current consumption in the Netherlands (van Rossum et al. 2020). The upper limit of food groups that did increase the risk of non-communicable diseases was based on the Eat-Lancet diet (Willett et al. 2019). In addition, a lower limit is placed on each food group if minimum consumption recommendations were available (Kromhout et al. 2016).

**Table 1:** Human nutrition related nutrients included in FOODSOM

Macronutrients	Fats	Micronutrients	Vitamins
Energy	Alpha linoleic acid	Potassium	Vitamin A (RAE)
Protein	Docosahexaenoic acid & eicosapentaenoic acid	Phosphorus	Vitamin B1
Dietary fibre	Linoleic acid	Calcium	Vitamin B2
Total fat	Cholesterol	Copper	Nicotinic acid
Carbohydrates		Iron	Vitamin B6
		Magnesium	Folate
		Sodium	Vitamin B12
		Zinc	Vitamin C
			Vitamin E
			Vitamin K

**Table 2:** Maximum and minimum consumption constraints of the current diet and the circular diet in grams per capita per day

Food group	Current	Circular	
		Minimum	Maximum
Grain	212	90	401^a^
Legume	5	9	40^a^
Tuber	88	0	239^a^
Fruit	129	200	345^a^
Vegetable	150	200	344^a^
Nut	15$	15	25^a^
Oil	31	0	47^b^
Sugar	40.4	0	31^b^
Meat	110	0	72^b^
Fish	16	14	100^a^
Dairy	362	0	500^b^
Egg	23	0	25^a^
Drink	0	0	500
Alcohol	138	0	138^b^

^a^Indicates constraints based on 95th percentile of current consumption (van Rossum et al. 2020)

^b^Indicates constraints based the EAT-Lancet diet (Willett et al. 2019)

^$^Indicates estimation using food-based dietary guidelines

### Greenhouse gas emissions

In FOODSOM, GHG emissions include emissions from the fertilisation of land, keeping of livestock, processing of food loss and waste and transport of products through the food system. GHG emissions related to the fertilisation of land and keeping of livestock are quantified using national GHG inventory methodologies or national emission modelling methodologies (Lagerwerf et al. 2019; van Bruggen et al. 2020). Nitrogen fertilisation of crops, N mineralisation from crop residues and peat soils results in N_2_O emissions. N_2_O emissions include direct and in-direct emissions with the latter resulting from the volatilisation of ammonia and nitrogen (di)oxide and the leaching of nitrate. The keeping of livestock contributes to methane (CH_4_), N_2_O and CO_2_ emissions. Livestock manure is a source of CH_4_ and N_2_O emissions and depended on feed intake. The emission factors applied to manure excretion were based on an average of current housing systems in the Netherlands (Lagerwerf et al. 2019; van Bruggen et al. 2020). Enteric fermentation by ruminants is a source of CH_4_ emissions and follows an IPCC Tier 2 approach.

Transporting crops, fish, food, by-products, manure and food waste resulted in CO_2_ emissions from the burning of fossil fuels. Distances from province to province (centre point to centre point) are quantified and the number of tonne kilometres quantified. Total tonne kilometres are multiplied by an emission factor for transportation (Blonk Consultants 2019). Capturing marine fish also resulted in GHG emissions from fishing boats; emission intensities are included per kg of fish caught (Rasenberg et al. 2013).

Composting food wasted resulted in N_2_O and CH_4_ emissions, emissions are based on the initial N and carbon content of food waste (i.e. the original compost feedstock) and the final N and carbon content (C:N ratio of 15) (Boldrin et al. 2009). To ensure food waste is suitable for livestock consumption, processing and sterilisation are required resulting in CO_2_ emissions (Salemdeeb et al. 2017; Silva Ortiz et al. 2020).

### Reference scenario

To test the accuracy and ability of FOODSOM to reproduce the current food system in the Netherlands, a reference scenario was developed. The objective of this reference scenario was to minimise the difference between the current diet and the modelled diet based on fixed (i.e. current, reference year 2017/2018) animal numbers, imports and exports and cultivated area of each crop. The current diet was taken from national food consumption surveys (van Rossum et al. 2020). The area of crop (e.g. hectares of wheat), number of animals (e.g. pigs), and quantity of food and raw materials imported and exported in the current food system was based on national statistics and food balance sheets (CBS 2019; FAO 2019). The diet composition and GHG emissions from the reference model are compared with available national statistics in the Supplementary Information.

### Food system designs

We examined the importance of three food system interventions leading to increased circularity: (1) reducing import and export of food, (2) dietary change, and (3) increased use of waste streams in the food system. The impact of import and export of food included three variants: (a) up to 50% of the nutrients in the human diet imported; (b) up to 25% of the nutrients in the human diet imported; and (c) no import (Table 3). To adhere to circularity principles, N, P, and K in imports and exports were balanced. This balance prevents nutrient surpluses/deficits in the Netherlands and other countries. Changes in human diets included two variants: (a) current diet; and (b) circular diet. As explained earlier, the current diet is based on current consumption patterns at a food group level. The circular diet alters consumption patterns to respect nutrient requirements and food group constraints to maintain a healthy and practical diet while reducing land use or GHG emissions. Furthermore, circular diets consider the interactions occurring between plant-sourced food production and animal-sourced food production (e.g. plant-sourced food production determines the by-products available to feed livestock, van Selm et al. (2022)) to realise the lowest possible agricultural land use and GHG emissions. Waste utilisation included two variants: (a) regulated use of waste streams and (b) full use of waste streams. Regulated use of waste streams allows current legal use of waste streams while full use of waste streams allows the feeding of livestock with food waste. Currently in the Netherlands, 16% of food loss and waste is fed to animals and 36% is composted (Soethoudt and Vollebregt 2020). The remaining (48%) is burnt or digested for electricity generation. In addition to food system interventions, the food system designs included two objectives: to either (a) minimise land use or (b) minimise GHG emissions. Multiplying each of these interventions and objectives leads to 3 × 2 × 2 × 2 = 24 food system designs. We assessed the effect of each of these interventions and objectives across all food system designs to show variation and sensitivity of the results.

**Table 3:** Overview and description of food system interventions

Theme	Intervention	Description
Import & export	No Import	No import implies a self-sufficient Netherlands
Import & export	50% Import	Up to 50% of each nutrient consumed in the human diet can be imported
Import & export	25% Import	Up to 25% of each nutrient consumed in the human diet can be imported
Dietary change	Current diet	Current diet is based on current consumption at a food group level in the Netherlands (e.g. grains, dairy) which enables substitution of specific food items within the food group (e.g. wheat for barley)
Dietary change	Circular diet	Circular diet alters consumption patterns to achieve minimum land use or GHG emissions. Nutrient restrictions (27 nutrients) ensure nutrient requirements are met and food group restrictions ensure the diet is practical and meets health recommendations
Waste use	Regulated waste use	Regulated waste use is based on the current legally permitted resource utilisation in the Netherlands (e.g. food waste cannot be fed to animals)
Waste use	Full waste use	Full waste use asses potential future resource utilisation whereby all waste streams can be utilised within a circular food system (e.g. food waste can then be fed to animals)
Objective	Minimise land	Minimise land, the objective function of the model is to minimise agricultural land use
Objective	Minimise GHG emissions	Minimise GHG emissions, the objective function of the model is to minimise GHG emissions

## Results

**Fig. 2:**
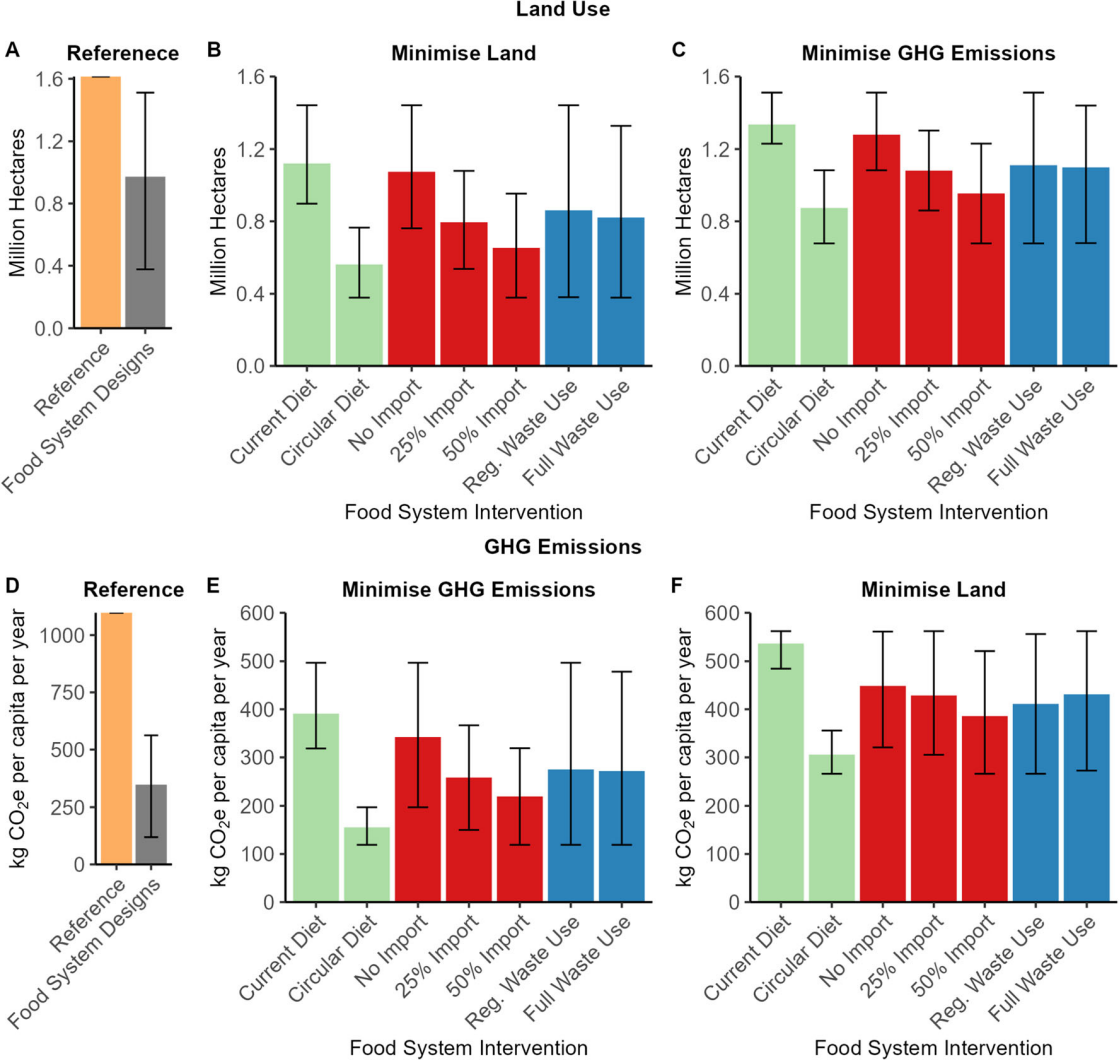
**a** Land use (in million hectares) in the Netherlands of the reference scenario and mean land use of the circular food system designs, **b** Land use (in million hectares) in the Netherlands when minimising land use per food system intervention, **c** Land use (in million hectares) in the Netherlands when minimising GHG emissions per food system intervention, **d** Total GHG emissions (carbon dioxide equivalent per capita (kg CO_2_e)) in the Netherlands of the reference scenario and mean total GHG emissions of the circular food system designs, **e** Total GHG emissions (carbon dioxide equivalent per capita (kg CO_2_e)) in the Netherlands when minimising land use from per food system intervention, **f** Total GHG emissions (carbon dioxide equivalent per capita (kg CO_2_e)) in the Netherlands when minimising GHG emissions per food system intervention. Bars indicate mean, range indicates minimum and maximum values of food system interventions

Our results show that land use and GHG emissions were on average 40% and 68% lower in the food system designs than in the reference scenario (Fig. 2). The reductions were primarily due to reduced production volumes in the Netherlands and optimising the choice of crops and animals. The food system in the Netherlands reduced the import of commodities (e.g. wheat, soy beans) and the export of food (e.g. pork, soy bean oil). Instead, the food system focused on feeding the domestic population using domestically produced food supplemented with imported food. On average, the N, P, and K in imports and exports decreased when comparing the results of the 24 food system designs to the reference scenario (Supplementary Information).

Within the food system designs, transitioning from the current diet to a circular diet was the most effective intervention to reduce land use and GHG emissions (43% & 52%) (Fig. 2). Allowing the import and export of food further reduced land use and GHG emissions in the Netherlands as domestically produced food was substituted for imported food which met the nutrients requirements of the population more efficiently (up to 34% & 26%). Allowing full use of waste streams had a minor impact on land use and GHG emissions (2% & 2%) (Fig. 2). Optimising the food system to minimise land use or GHG emissions already prioritised the re-use and recycling of food loss and by-products in the food system.

### Reference scenario

**Fig. 3:**
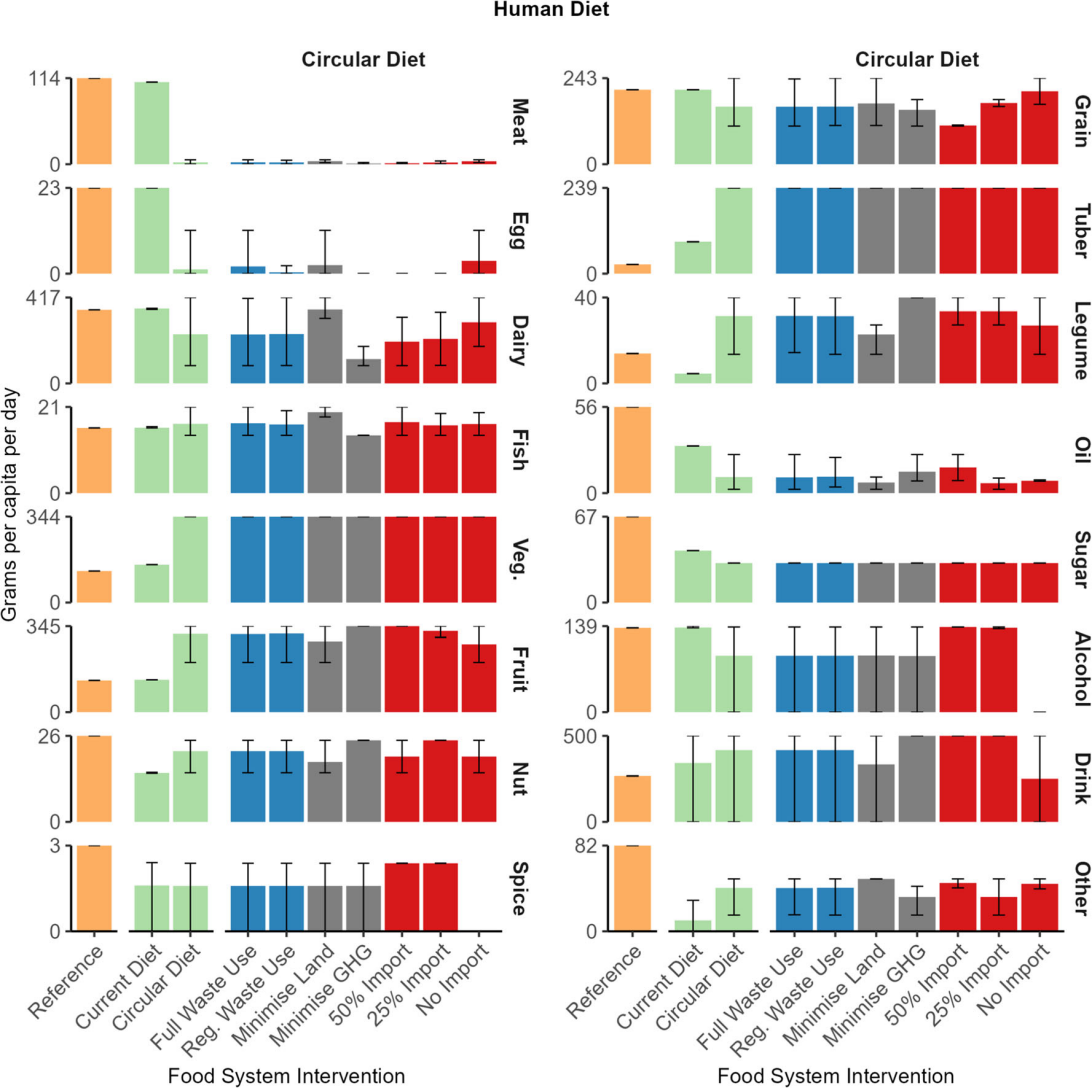
Human consumption in grams per capita per day per food group of each food system intervention. Bars indicate mean, and range indicates minimum and maximum values of food system interventions

The dietary composition of our modelled reference scenario shows some differences with the current Dutch diet (Fig. 3; Supplementary Information). Discrepancies between the current diet and the modelled reference diet can be explained by uncertainty in food loss and waste fractions which impacted the modelled reference diet. In addition, imports and exports were fixed based on FAO food balance sheets, which also impacted the modelled reference diet. Emissions were lower in the reference scenario of this study compared to the Dutch national emission inventory (RIVM 2020) (Supplementary Information).

### Human diets

The circular diet intervention reduced land use on average by 43% and GHG emissions on average by 52% compared to the current diet (Fig. 2). The circular diet optimised food production and consumption simultaneously to minimise land use or GHG emissions across the entire food system. Human dietary requirements were firstly met with plant-sourced food while animal-sourced food was only included to meet the remaining essential nutrients. Notable increases in consumption in the circular diet included fruit (315 vs. 130 g per capita per day), vegetable (344 vs. 152), legume (31 vs. 5), tuber (239 vs. 89), and nut (22 vs. 15) food groups, while notable decreases in the circular diet included meat (2 vs. 109), oil (10 vs. 30), egg (1 vs. 23), and sugar (31 vs. 41) food groups compared to the current diet. Notably, a decreased consumption of meat, oils, and sugar in the circular diet will also lead to better health outcomes with reduced risk of non-communicable diseases (e.g. colorectal cancer, cardiovascular disease and type-2 diabetes) (Godfray et al. 2018; Springmann et al. 2018; Willett et al. 2019).

In the circular diet, consumption of the alcohol, grain and spice food groups was driven by the import food system interventions (Fig. 3). Consumption of alcohol and spice decreased, while consumption of grains increased when imports were prevented. The alcohol and spice food groups only included consumption of food items that could not be produced in the Netherlands, e.g. wine. Lastly, banning imports increased consumption of grains (50% import: 110 g per capita per day, no import: 206) to compensate for the lack of imported food groups.

In the circular diet, consumption of the following food groups was driven by the food system objective (i.e. either minimise land use or minimise GHG emissions): dairy, fish and legume (Fig. 3). Consumption of the legume food was greater when minimising land use than when minimising GHG emissions: fish (20 vs. 14 g per capita per day) and dairy (360 vs. 118). However, consumption of legumes (40 vs. 23) and oil (14 vs. 7) was greater when minimising GHG emissions. Lower N use of legume crops favoured the legume food group when minimising GHG emissions. Dairy cows produce methane, which decreased consumption of dairy foods when minimising GHG emissions.

Consumption of the vegetable food groups was always close to the maximum permitted consumption in the circular diet. In addition, the waste use interventions had little influence on the human diet (Supplementary Information).

### Imports and exports

The 50% import intervention decreased domestic land use on average by 34% and GHG emissions on average by 26% compared to the no import intervention (Fig. 2). In comparison, the 25% Import intervention decreased domestic land use on average by 21% and GHG emissions on average by 15% compared to the no import intervention. The mitigating effect of import on land use and GHG emissions is explained by the fact that domestically produced food items were substituted with imported alternatives. Food items were exchanged based on N, P, and K contents and land use efficiency (i.e. yield) or GHG emission intensity. In addition, the inclusion of imported food items allowed the diet to more efficiently meet the precise dietary requirements of the Dutch population, which reduced over consumption of nutrients. For example, with the circular diet, the intake of essential fatty acids EPA and DHA was ‘only’ 49% above minimum nutrient requirements when imports were allowed import compared to 65% in the no import intervention.

**Fig. 4:**
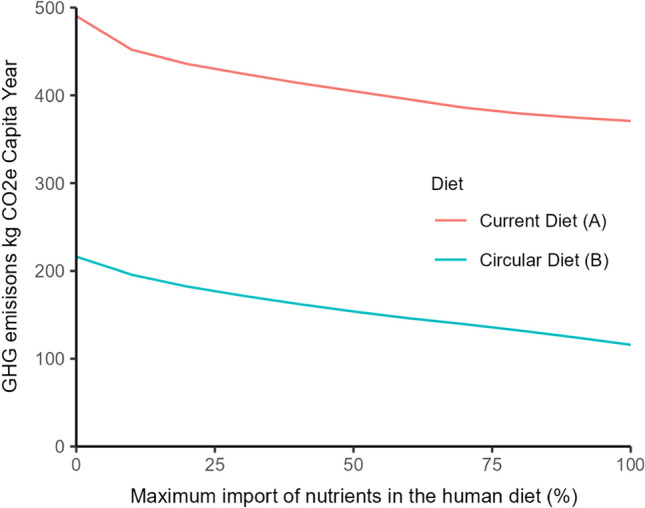
Percentage of the human diet imported in 10% increments (0–100%) and total GHG emissions in the Netherlands (carbon dioxide equivalent per capita (kg CO_2_e). Line **A** is a food system design with a current diet, full waste use and minimum GHG emissions, **B** is a food system design with a circular diet, full waste use and minimum GHG emissions

Increasing the import of food leads to more imports and exports into the Netherlands, but the overall GHG mitigation potential decreases as more food is imported and exported (Fig. 4). For example, the 50% and 25% import intervention decreased GHG emissions by 26% and 15%, respectively, compared to the no import intervention. The initial import (i.e. 25% import) of food reduced impact on land use and GHG emissions the most.

**Fig. 5:**
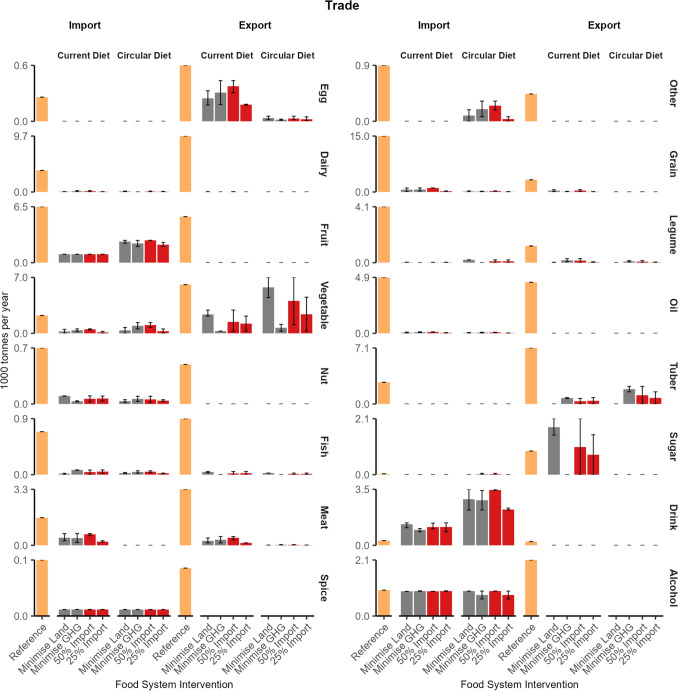
Trade per food family in thousand tonnes per food system intervention. Bars indicate mean, and range indicates minimum and maximum values of food system interventions

Imports of the following food groups were driven by the human diet interventions: meat, fish, fruit, drink, legume, and other (Fig. 5), because of the differences in consumption between the current diet and the circular diet (Fig. 3). Imports of the meat and fish food groups were greater in the current diet while imports of the fruit, drink, legume, and other food groups were greater in the circular diet. The import of remaining food groups was driven by a combination of import interventions, diet interventions and food system objectives.

When importing food items, food exports were necessary to balance the N, P, and K imported. The balance ensured that there is no accumulation or depletion of nutrients between the global food system and the Netherlands. Exports of the following food groups were driven by the human diet: egg, meat and dairy (Fig. 3). The quantity of exports was greater in the current diet intervention due to more imports. Eggs, fish, dairy and meat were exported to balance nutrients in imported meat and dairy. Exports of the vegetable, legume and sugar (current diet only) food groups were driven by the food system objective. Optimising for land use increased the export of the vegetable and sugar food groups, while optimising for GHG emissions increased the export of crops in the legume food group. Exported vegetables included cabbages, cucumbers, aubergines and mushrooms. Cucumbers, aubergines and mushrooms are grown in greenhouse and mushroom sheds with high yields and minimal land use.

### Livestock

**Fig. 6:**
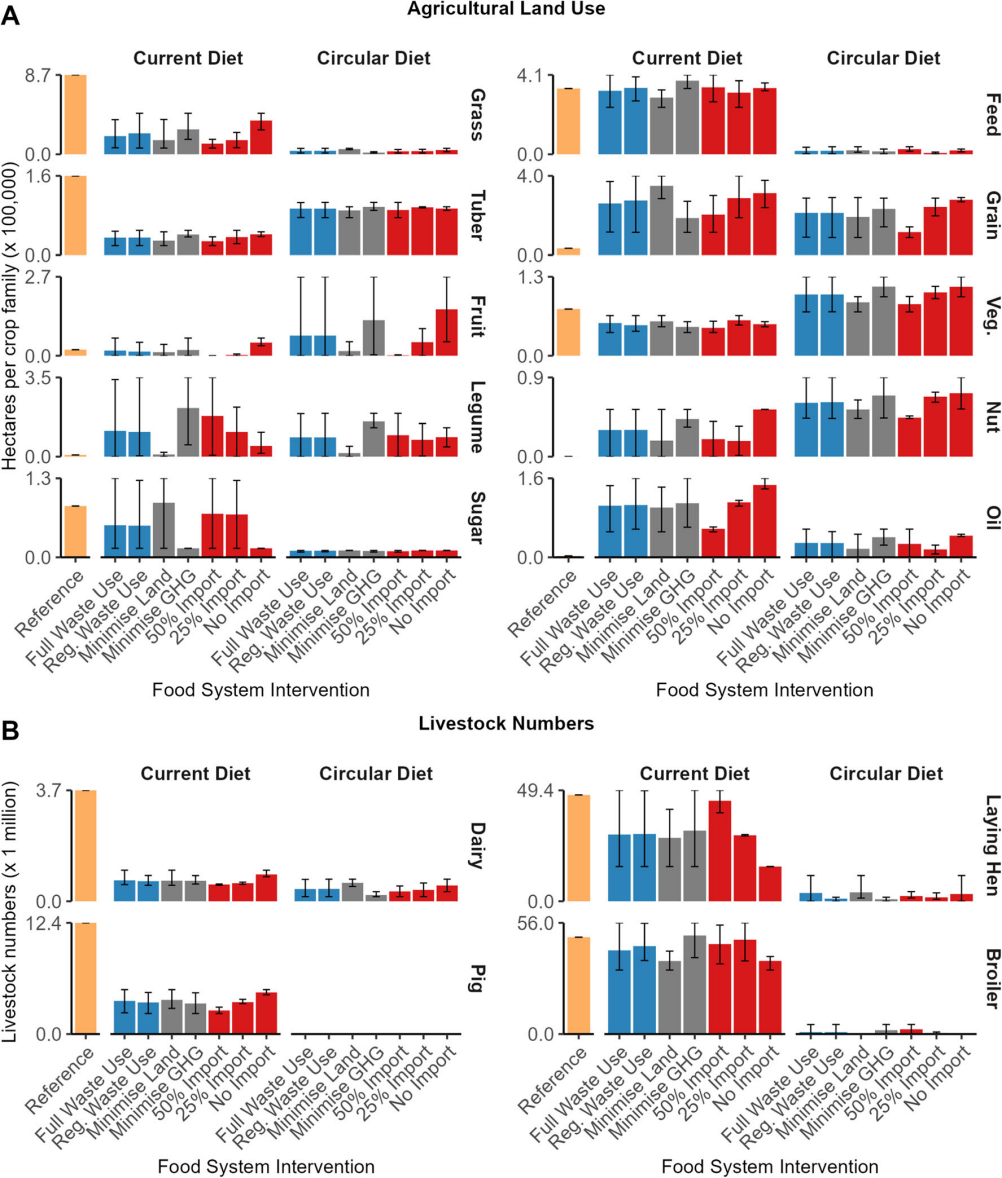
**a** Cropland area in hectares per crop family of each food system intervention. Grains, legumes, and oil groups are for food production. Grains, legumes, and oil for feed production belong to the feed group, **b** total livestock numbers per animal production system of each food system intervention. Bars indicate mean, range indicates minimum and maximum values of food system interventions

Livestock numbers in all livestock categories were lower in the circular diet than in the current diet due to reduced consumption of livestock products (Figs. 3 and 6b). With the current diet, livestock numbers in the following livestock categories were driven by the import interventions: pigs, laying hens, and dairy cows The number of laying hens increased in the 50% import intervention due to an increase in egg exports (eggs were produced to balance nutrients of imported animal-sourced food). At the same time, the number of pigs decreased in the 50% import intervention due to increased meat imports (Fig. 5). With the circular diet, livestock numbers in the following livestock categories were driven by the food system objective: dairy cows, laying hens, and broilers. The number of broiler chickens increased when minimising GHG emissions, while the number dairy cows and laying hens increased when minimising land use emissions. Broiler chickens have a low GHG emission intensity but require high-quality feed ingredients, for example, 90% of the broiler diet was high-quality co-products in the circular human diet (due to low broiler number and meat requirements in the circular diet). Dairy cows utilise fresh grass, grass silage and hay which resulted in the lowest land use in the model. However, dairy cows also produce methane, which increased GHG emissions (Fig. 2).

Feeding food waste to livestock in the full waste use intervention decreased land use on average by 2% and increased GHG emissions on average by 2% (Fig. 2). Processing food waste into animal feed (e.g. sterilising, drying) is emission intensive which resulted in additional GHG emissions (Salemdeeb et al. 2017), thereby limiting the GHG emission mitigation potential of the full waste use intervention. Full waste use intervention had little impact on the number and types of livestock kept.

### Land use

Land use in the following crop categories was driven by the human diet: animal feed, grassland, oil crops, sugar, tuber, fruit and vegetables (Fig. 6a). The land area of animal feed, grassland, sugar and oil crops was greater in the current diet while the land area of tuber, fruit and vegetables was greater in the circular diet. These changes in land area were in line with changes in consumption patterns between the circular and current diet (Fig. 3). Land use in the following crop categories was driven by the import intervention: grassland (current diet), oil crops (current diet), grain (circular diet), legumes (circular diet) and nut trees (circular diet). Preventing imports increased the domestic land area of these crop categories.

Land use in the following crop categories was driven by the food system objective: legume (both diets) and grain (current diet only) (Fig. 6a). Minimising GHG emissions increased the area of legume crops while minimising land increased the area of grain crops. Legume crop fixed N, which reduced the demand for artificial fertiliser and production-related GHG emissions when minimising GHG emissions. Finally, the area of grains for human consumption and feed crops was sensitive to the proportion of grain yield suitable for human consumption. Reducing the grain yield suitable for human consumption increased the area of grains for human consumption (i.e. the share of grains in the diet hardly changed) and decreased the area of feed crops (Supplementary Information).

## Discussion

Our analysis focused on exploring the role of imports and exports, dietary change and waste utilisation as interventions to reduce land use and GHG emissions in a circular food system. However, other interventions could also play a role, for example, the productivity level and associated N use efficiency of crop production could further improve nutrient cycling and reduce GHG emissions (Silva et al. 2021). The transition to a circular food system will also alter management practices other than fertilisation (De Boer and Van Ittersum 2018), which were not considered in this analysis (e.g. reduced pesticide use). Reducing the total production volume by reducing imports to and exports from the Netherlands and shifting to a more circular food system resulted in a substantial reduction in land use at a national level. The reduction in land use could provide space for somewhat lower crop yields requiring more land and different management practices. Lower crop yields could have potential benefits for N use efficiency and on-farm biodiversity (Kleijn et al. 2009). Alternatively, surplus land can be used for reforestation to partially offset GHG emissions of food production (Doelman et al. 2020).

Land use results from our food system designs were different compared to land use estimates of current Dutch consumption. Nijdam et al. (2019) found 3.2 million hectares of land was required for current Dutch food consumption. This is substantially greater than the 1.2 million hectares in our food system designs with a current diet, no imports and regulated waste use. Nijdam et al. (2019) multiplied current consumption patterns, crop yields and life cycle assessment estimates for land use of food items into total land use of Dutch consumption. In contrast, our study was optimised to minimise land use or GHG emissions. Consumption in the current diet interventions was equal to current consumption at a food group level, but individual food items within a food group could vary. This allowed food items with the lowest land use or GHG emission to be selected within the food group. Moreover, we applied a food system approach which accounted for relationships between products in the food system (e.g. wheat producing flour for human consumption and wheat bran for animal consumption) and modelled livestock diets for the lowest land use or GHG emissions. Another study with a similar method as ours found 0.9 million hectares of land was required for the Dutch consumption if all imports and exports were halted (Terluin et al. 2013).

Our results show a strong reduction in production volumes in the Netherlands, the reduction in animal-sourced food production and export could have an influence on global food security. However, livestock in the Netherlands are fed with a substantial amount of imported feed, which requires arable land; this arable land could be used to produce food instead of feed and therefore contribute to global food security (Mottet et al. 2017; van Grinsven et al. 2019). This would require an increase in plant-sourced food consumption and a decrease in animal-sourced food consumption in recipient countries, potentially leading to healthier diets, better health outcomes, and increased food security. Improving the distribution of food (i.e. less food products consumed by livestock) may create a more just food system, but consuming less animal-sourced food also has cultural implications not considered in this study.

In this study, we look at the optimal distribution of food without considering the implications on food sovereignty. The shift in the Netherlands towards more local production supplemented with imported food may strengthen peoples ties to the food system and especially food producers (Enthoven and Van den Broeck 2021). A more local or self-sufficient food system may reduce the power of large global actors (IPES-Food 2017). On the other hand, it also reduces dietary diversity. Similarly, other factors that influence supply and demand in the food system including, e.g. economics, policies, dietary preferences and other cultural aspects were not included in this analysis. Our research explores the potential environmental benefits and configurations of a more circular food system. Societal changes needed to enable a transition is a valuable avenue for further research, we hypothesise that practical implementations will most likely require consorted governmental policies.

We assumed all by-products and food waste should be used as animal feed or applied to the soil as an organic amendment. The transition towards increased circularity is a societal shift, not necessarily limited to the food system (Muscat et al. 2021). There will also be demand for waste streams and biomass outside the food system (e.g. bio-energy, bio-plastics) creating competition with the food system (Stegmann et al. 2020). Assessing which waste streams and biomass will be valuable for different parts of society requires an expansion of FOODSOM to other economic sectors. Utilising food waste as animal feed only reduced GHG emissions by 2%, therefore utilising food waste for, e.g. energy production may be more effective at reducing societies total GHG emissions.

FOODSOM is a linear optimisation model implemented for the Netherlands. Because the model has been specifically applied to the Netherlands imports and exports are not linked to a country of origin or country of consumption. The decision to export products was not determined by demand of the products end consumers. Instead, in the model, the decision to export was driven by the model constraint to balance in- and export of N, P, and K from the Netherlands. The disadvantage of using a nutrient balance is that food items with a low N, P, and K content (e.g. sugar, potato starch, oils) could be imported rather abundantly, compensated by smaller export quantities with relatively high nutrient concentrations. In addition, the nutrient balance does not account for the land use and GHG emissions from imported products, instead land use and GHG emissions from exported products were assigned to the Netherlands. This could result in an imbalance of land use and GHG emissions. In practice, the decision to import or export would evidently be set by the importing or exporting countries. Finally, the (environmental) implications for importing and exporting animal feed in the Netherlands could also be explored using a model covering a larger geographical area. On the other hand, expanding the model may lead to a loss of detail and precision due to data availability (e.g. crop yields).

In reality, future food systems will never be optimised for one environmental objective (e.g. GHG emissions, land use), instead a compromise between environmental objectives may be needed or priority given to environmental objectives with the highest degree of urgency. In this study, GHG emissions were on average 27% higher when minimising land, while land use was on average 71% higher when minimising GHG emissions (Fig. 6). Economics will also always play a role in the design of the food system. Therefore, these results may present an overly optimistic perspective on potential land use and GHG mitigation. This study is very much of an explorative nature (Van Ittersum et al. 1998) and, therefore, does not aim to make any predictions (Van Ittersum et al. 1998). Yet, the outcomes do improve our understanding of interrelationships in the food system, waste use and environmental impacts and which interventions in production and consumption will be most effective in reducing environmental impacts in a circular food system. This can give guidance to shaping and transitions to circular food systems in the Netherlands and beyond.

## Conclusion

Our results show that creating a more circular food system requires substantial changes. Achieving a circular diet requires changing people’s consumption behaviours to reduce meat consumption and increase consumption of, e.g. vegetables and legumes. Thus, specifically in the Netherlands, changes in agricultural land use are required to prioritise food crops over animal feed crops. The number of livestock would need to decrease under the assumption of no imports of animal feed and a limited export of livestock products.

**Fig. 7:**
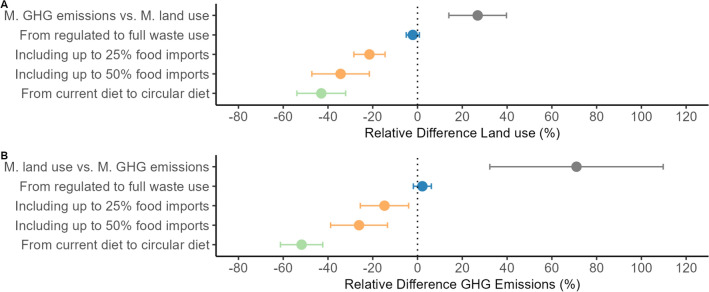
**a** Mean relative difference in land use between different paired food system interventions, e.g. 25% import compared to no import across all relevant food system designs, **b** mean relative difference in GHG emissions between different food system interventions, e.g. full waste use compared to regulated waste use. Ranges indicate standard deviation of food system interventions paired comparisons between food system designs

We show how different food system interventions to increase circularity can reduce land use and GHG emissions in the Dutch food system. Land use and GHG emissions resulting from the food system designs were on average 40% and 68% lower than in the current food system, primarily driven by a reduction in production volumes and a shift towards feeding the domestic population. Within the food system designs, shifting from the current diet to a circular diet was the most effective intervention to reduce the land use and GHG emissions (by, respectively, 43% & 52%) (Fig. 7). The circular diet altered consumption patterns to achieve minimum land use and GHG emissions. Maintaining limited trade in a circular food system in combination with a nutrient balance of imports and exports also contributed to a reduction of land use and GHG emissions in the Netherlands (by, respectively, up to 34% & 26%). Domestically produced food was substituted with imported alternatives (resulting in net zero nitrogen, phosphorus and potassium exchange). Circularity interventions should not be implemented mutually exclusively; by combining a circular diet supplemented with imported food and full waste interventions, the lowest land use and GHG emissions can be realised. (Supplementary Information).

### Supplementary Information

Below is the link to the electronic supplementary material.

Supplementary file1 (PDF 848 kb)

## Data Availability

All original code and required data have been deposited in a git repository and are publicly available: https://git.wur.nl/aps/foodsom-public. Additional model explanation is included in the repository.
