# Supplementary material for: Interventions to increase circularity and reduce environmental impacts in food systems
Source: Ambio. 2023 Nov 16;53(3):359–75. doi: 10.1007/s13280-023-01953-x (PMC10837400; doi:10.1007/s13280-023-01953-x)
Supplement: Supplementary file 1 — Supplementary file1 (PDF 848 kb) [file 13280_2023_1953_MOESM1_ESM.pdf]

Ambio

Supplementary Information
This supplementary information has not been peer reviewed.

Title: **Interventions to increase circularity and reduce environmental impacts in food systems**

Authors: Benjamin van Selm, Hannah H.E. van Zanten, Renske Hijbeek, Corina E. van Middelaar, Marijke Schop, Martin K. van Ittersum, Imke J.M. de Boer

Supplementary Information

Supporting Reference Scenario Results

To test the accuracy and ability of FOODSOM to reproduce the current food system in the Netherlands a reference scenario was created. The objective of the reference scenario was to minimise the difference between the current diet and the modelled diet based on fixed (i.e., current, reference year 2017/2018) animal numbers, imports and exports and cultivated area of each crop.

A small difference between the current diet and the modelled diet indicates a high ability of FOODSOM to reproduce the current food systems in the Netherlands (Figure S1). Similarly, a small difference between total GHG emissions from the reference scenario and current national estimates also indicates a high ability of FOODSOM to model environmental impacts of the Dutch food system. Results from the modelled reference diet and the current diet are shown in table S1. Discrepancies between the current diet and the modelled diet can be explained by uncertainty in food loss and waste factions which impacted the modelled reference diet. In addition, imports and exports were based on FAO food balance sheets, which also impacted the modelled reference diet (e.g., nuts, legumes).

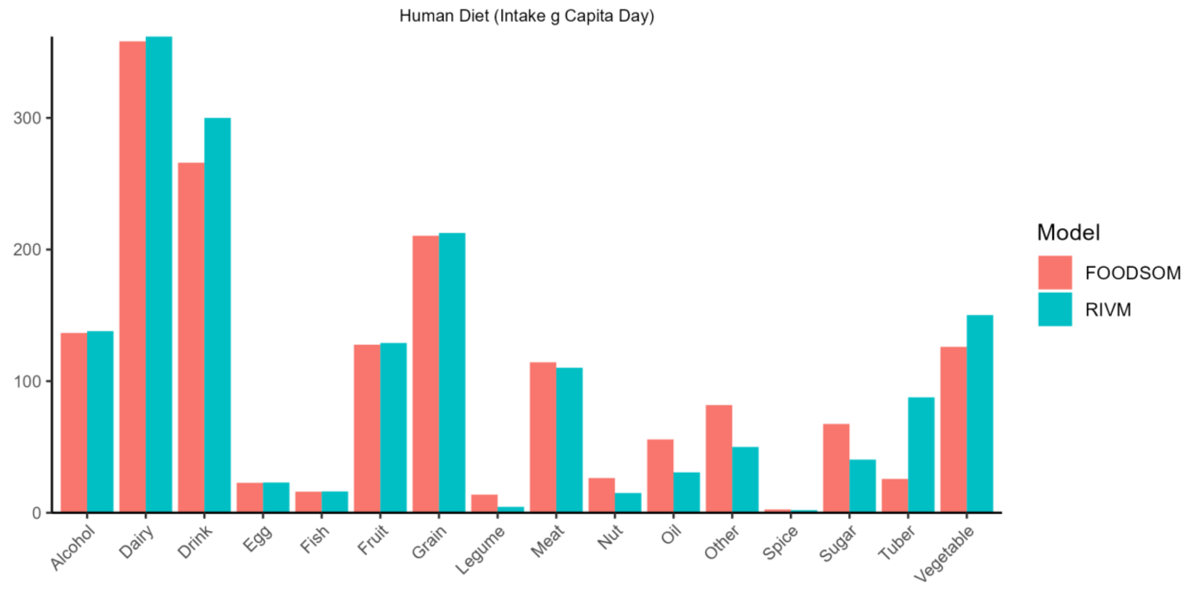

Figure S1: Modelled reference diet compared to the current diet.

Figure S2 shows acidifying nitrogen emissions and GHG emissions from the reference scenario compared to results from the national emission model (NEMA) (Van Bruggen et al. 2022). Emission results from FOODSOM were lower than NEMA due to lower nitrogen (N) intake and excretion from livestock in FOODSOM (small ruminants were also not included in FOODSOM). In FOODSOM, feed intake is determined by the feed available, which is based on crop yields, and imports and exports. Feed intake is then multiplied by N content from feed nutrient databases to calculate N intake (Spek and Van Wesemael 2021). The NEMA model approaches livestock diets differently to FOODSOM, livestock

diets and N contents are collected from the feed industry to calculate nitrogen intake and excretion. The difference in approaches is likely to explain why N excretion was lower in FOODSOM and corresponding emissions from livestock and fertilisation are also lower.

The FOODSOM and NEMA approach to estimate methane emissions is also different. NEMA uses a tier three approach for methane emissions for dairy cattle. FOODSOM however uses a tier two approach, insufficient data was available for food losses as animal feed to include a tier three approach in the FOODSOM model.

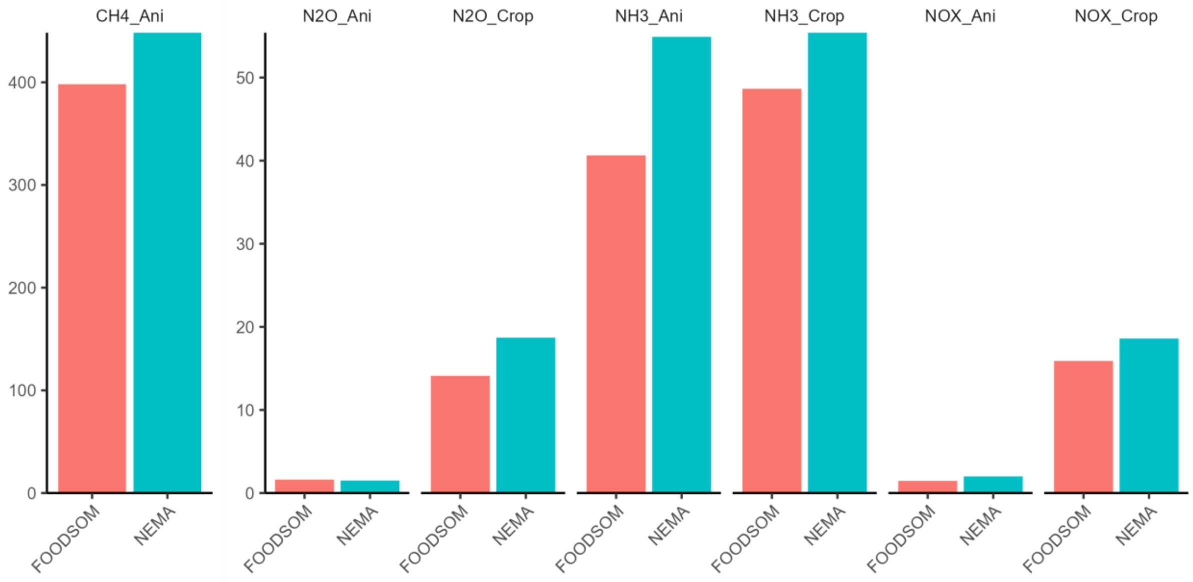

Figure S2: Acidifying nitrogen emission results and GHG emission results from the reference scenario (FOODSOM; this article) and the national emission model (NEMA; Van Bruggen et al. (2022)). Results shown in Gg.

Supporting Grain Yield Results

Currently in the Netherlands grains (especially wheat) are predominantly produced for animal consumption. However, in this analysis we also allowed grains in the Netherlands to be produced for human consumption. Grains for human consumption have a higher quality standard, largely due to bread making requirements. In order to assess the implications of these requirements we performed an additional sensitivity analysis. Firstly, all grains were assumed suitable for human consumption without a yield penalty (assumption applied in this study). Secondly, the yield of grains for human consumption was reduced by 15% and only 50% of the yield was suitable for human consumption. The remaining 50% was fed to livestock; thus it was assumed not all grains produced in a given year would be suitable for human consumption. The land area of grains for food consumption was on average 52% higher in the food system designs when only 50% of the grain yield could be used for human consumption. Simultaneously the area of feed production decreased by 39% in the food system designs as less feed

specific crops were required. Finally, total land use increased by on average 9% and GHG emissions increased by on average 8% .

Supporting Food System Design Results

Table S1: Overview of food system designs codes and associated food system interventions.

Scenario	Diet	Waste Use	Import	Optimisation
Reference	Reference	Reference	Reference	Reference
Cu_RW_50_ML	Current Diet	Reg. Waste Use	50% Import	Minimise Land
Cu_RW_50_MG	Current Diet	Reg. Waste Use	50% Import	Minimise GHG
Cu_RW_25_ML	Current Diet	Reg. Waste Use	25% Import	Minimise Land
Cu_RW_25_MG	Current Diet	Reg. Waste Use	25% Import	Minimise GHG
Cu_RW_0_ML	Current Diet	Reg. Waste Use	No Import	Minimise Land
Cu_RW_0_MG	Current Diet	Reg. Waste Use	No Import	Minimise GHG
Cu_FW_50_ML	Current Diet	Full Waste Use	50% Import	Minimise Land
Cu_FW_50_MG	Current Diet	Full Waste Use	50% Import	Minimise GHG
Cu_FW_25_ML	Current Diet	Full Waste Use	25% Import	Minimise Land
Cu_FW_25_MG	Current Diet	Full Waste Use	25% Import	Minimise GHG
Cu_FW_0_ML	Current Diet	Full Waste Use	No Import	Minimise Land
Cu_FW_0_MG	Current Diet	Full Waste Use	No Import	Minimise GHG
Ci_RW_50_ML	Circular Diet	Reg. Waste Use	50% Import	Minimise Land
Ci_RW_50_MG	Circular Diet	Reg. Waste Use	50% Import	Minimise GHG
Ci_RW_25_ML	Circular Diet	Reg. Waste Use	25% Import	Minimise Land
Ci_RW_25_MG	Circular Diet	Reg. Waste Use	25% Import	Minimise GHG
Ci_RW_0_ML	Circular Diet	Reg. Waste Use	No Import	Minimise Land
Ci_RW_0_MG	Circular Diet	Reg. Waste Use	No Import	Minimise GHG
Ci_FW_50_ML	Circular Diet	Full Waste Use	50% Import	Minimise Land
Ci_FW_50_MG	Circular Diet	Full Waste Use	50% Import	Minimise GHG
Ci_FW_25_ML	Circular Diet	Full Waste Use	25% Import	Minimise Land
Ci_FW_25_MG	Circular Diet	Full Waste Use	25% Import	Minimise GHG
Ci_FW_0_ML	Circular Diet	Full Waste Use	No Import	Minimise Land
Ci_FW_0_MG	Circular Diet	Full Waste Use	No Import	Minimise GHG

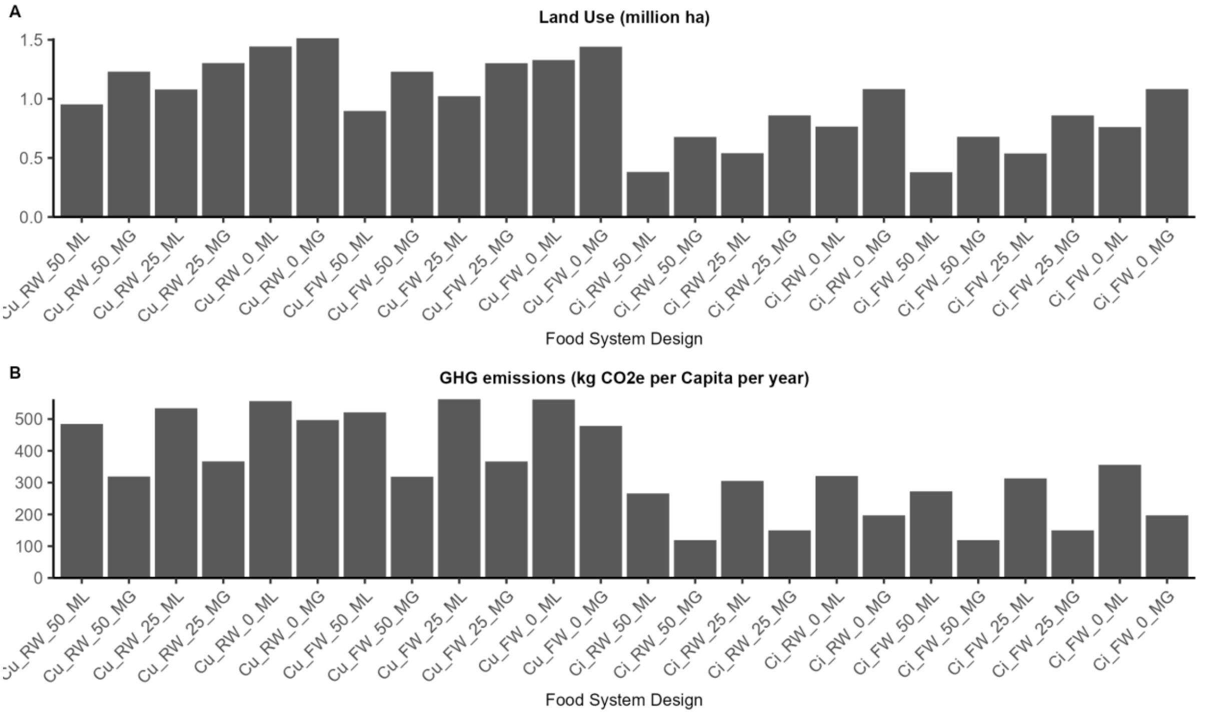

Figure S3: A, Land-use in million hectares per food system design; B, GHG emissions from cropland and livestock in kg CO2e per capita per food system design

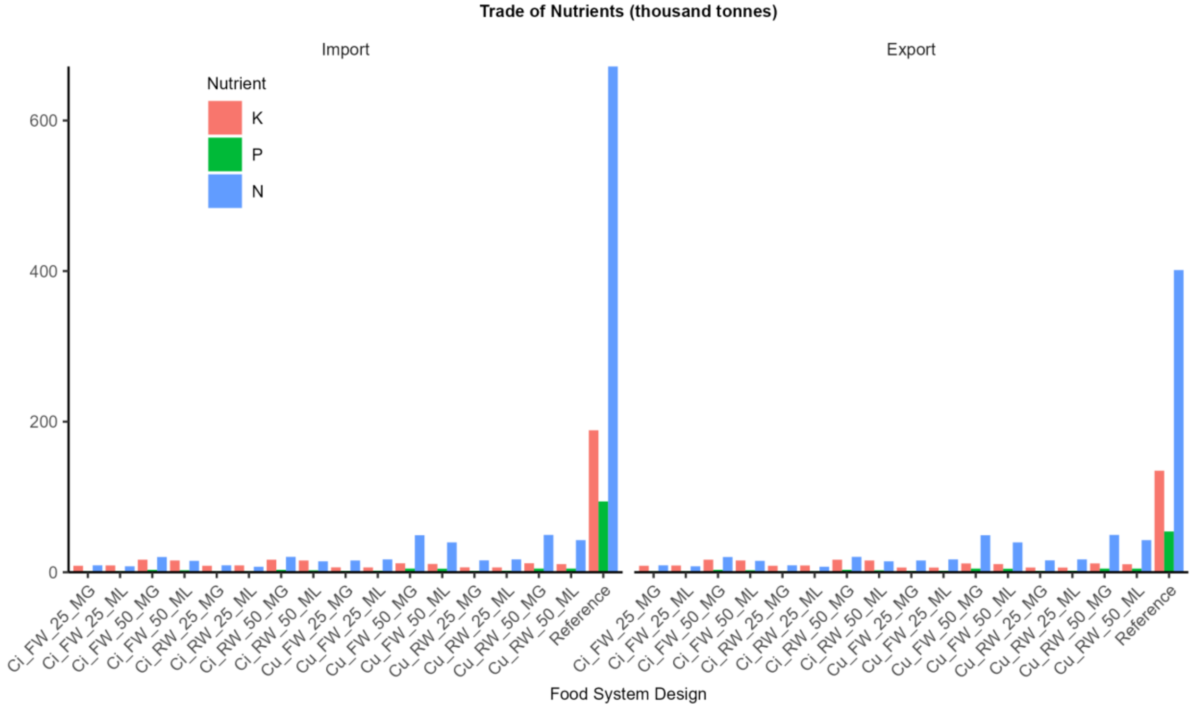

Figure S4: Trade of nutrients in thousand tonnes per food system design.

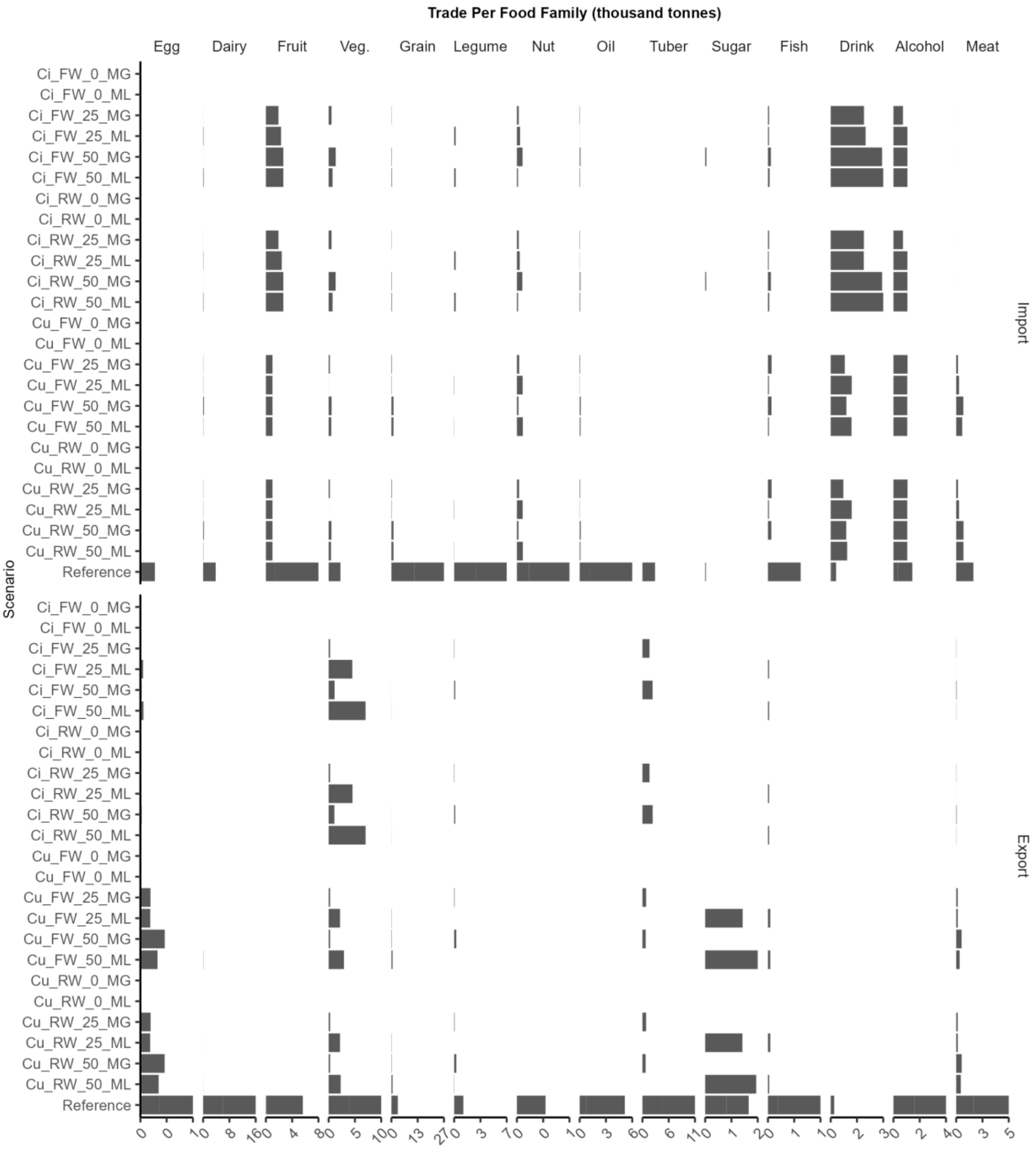

Figure S5: Trade in thousand tonnes per food family per food system design

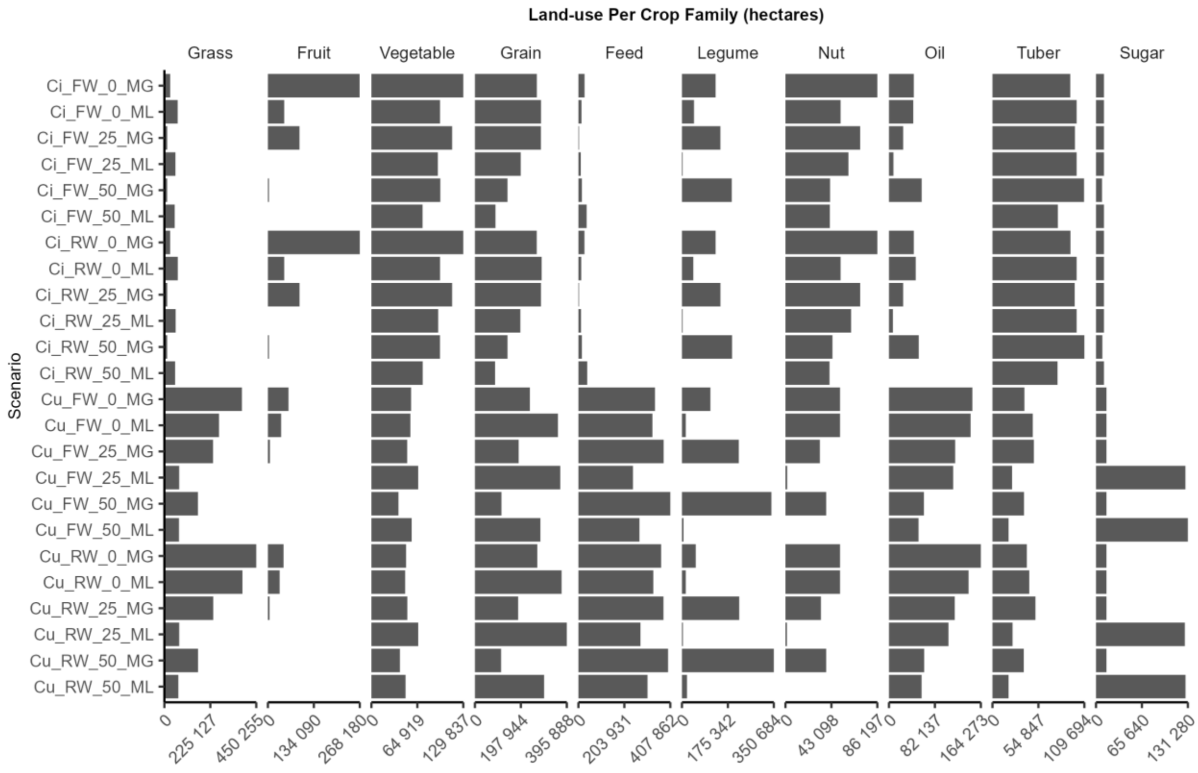

Figure S6: Land-use per crop family per food system design.

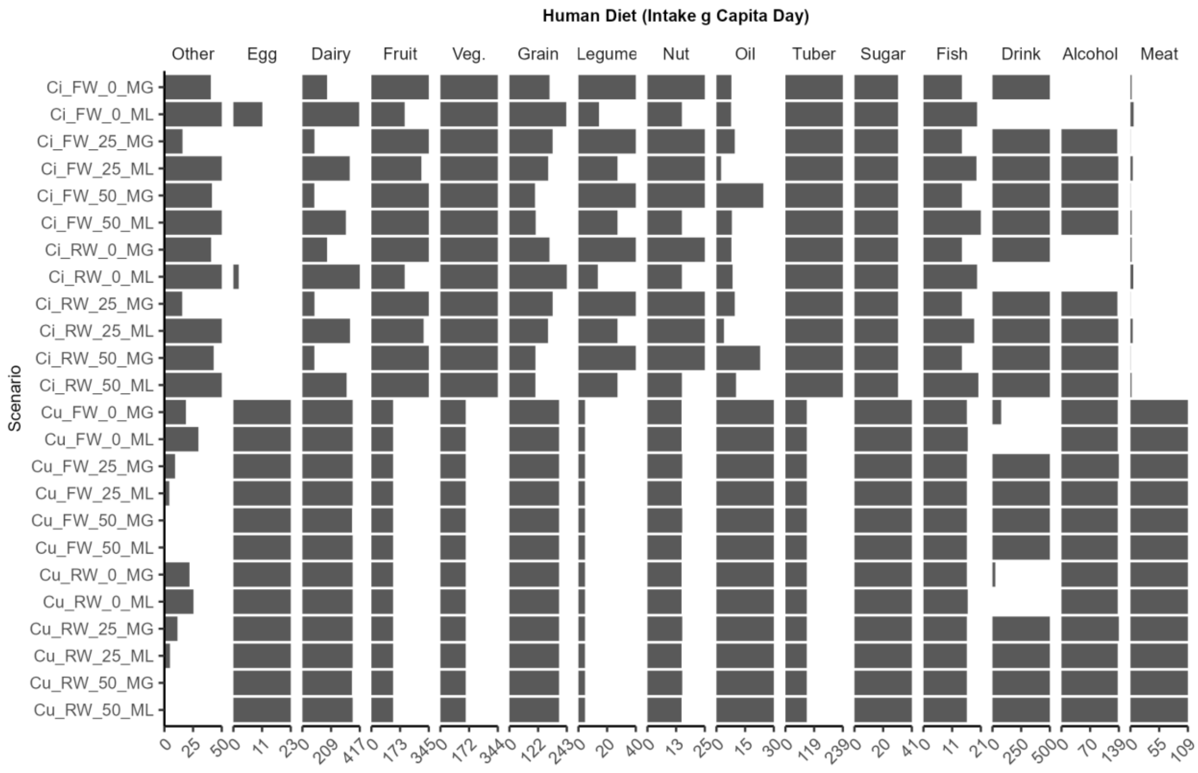

Figure S7: human diet in grams per capita per day per food system design.

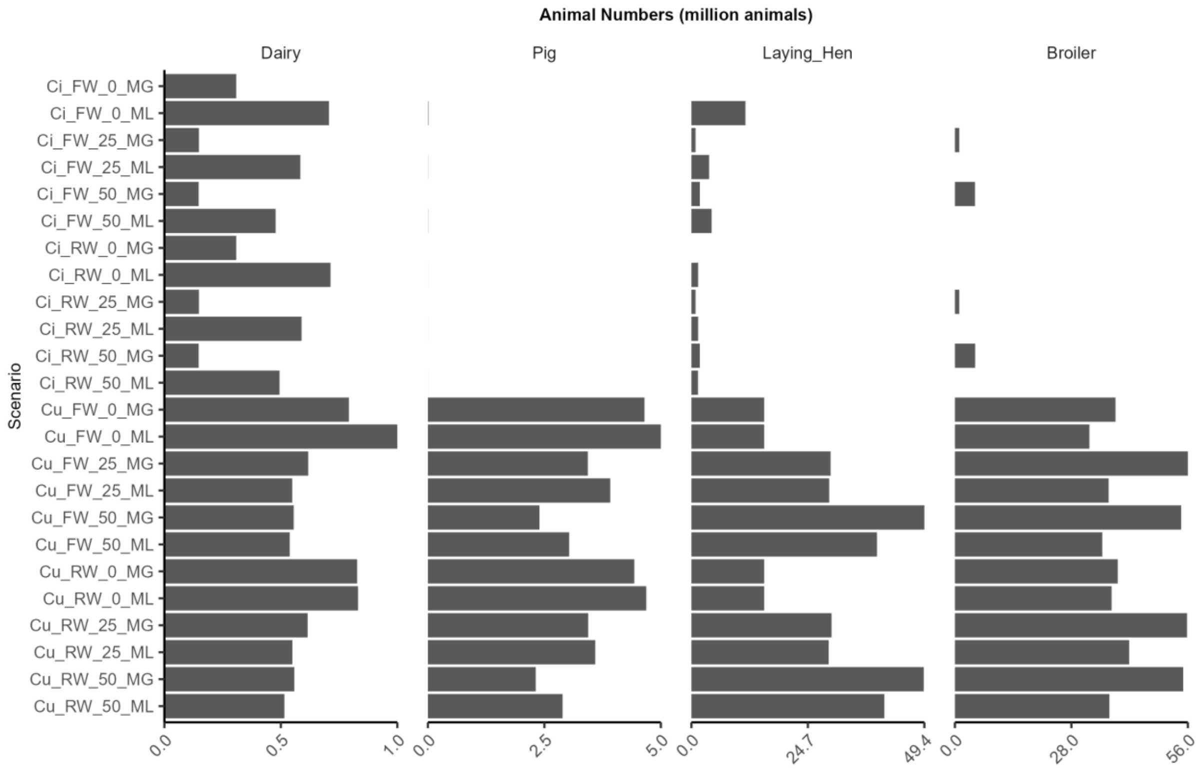

Figure S8: Animal numbers per food system design.
